# Novel Insights Into the Potential Mechanisms of N6-Methyladenosine RNA Modification on Sepsis-Induced Cardiovascular Dysfunction: An Update Summary on Direct and Indirect Evidences

**DOI:** 10.3389/fcell.2021.772921

**Published:** 2021-11-12

**Authors:** Yang Wang, Miaomiao Xu, Peng Yue, Donghui Zhang, Jiyu Tong, Yifei Li

**Affiliations:** ^1^ Key Laboratory of Birth Defects and Related Diseases of Women and Children of MOE, Department of Pediatrics, West China Second University Hospital, Sichuan University, Chengdu, China; ^2^ Department of Immunology, West China School of Basic Medical Sciences and Forensic Medicine, Sichuan University, Chengdu, China; ^3^ State Key Laboratory of Biocatalysis and Enzyme Engineering, School of Life Science, Hubei University, Wuhan, China

**Keywords:** m6A modification, sepsis, myocardial injuries, mitochondrial damages, inflammation response

## Abstract

Sepsis is a life-threatening organ dysfunction caused by a host’s dysfunctional response to infection. As is known to all, septic heart disease occurs because pathogens invading the blood stimulate the activation of endothelial cells, causing a large number of white blood cells to accumulate and trigger an immune response. However, in severe sepsis, the hematopoietic system is inhibited, and there will also be a decline in white blood cells, at which time the autoimmune system will also be suppressed. During the immune response, a large number of inflammatory factors are released into cells to participate in the inflammatory process, which ultimately damages cardiac myocytes and leads to impaired cardiac function. N6-methyladenosine (m6A) is a common RNA modification in mRNA and non-coding RNA that affects RNA splicing, translation, stability, and epigenetic effects of some non-coding RNAs. A large number of emerging evidences demonstrated m6A modification had been involved in multiple biological processes, especially for sepsis and immune disorders. Unfortunately, there are limited results provided to analyze the association between m6A modification and sepsis-induced cardiovascular dysfunction (SICD). In this review, we firstly summarized current evidences on how m6A mediates the pathophysiological process in cardiac development and cardiomyopathy to emphasize the importance of RNA methylation in maintaining heart biogenesis and homeostasis. Then, we clarified the participants of m6A modification in extended inflammatory responses and immune system activation, which are the dominant and initial changes secondary to sepsis attack. After that, we deeply analyzed the top causes of SICD and identified the activation of inflammatory cytokines, endothelial cell dysfunction, and mitochondrial failure. Thus, the highlight of this review is that we systematically collected all the related potential mechanisms between m6A modification and SICD causes. Although there is lack of direct evidences on SICD, indirect evidences had been demonstrated case by case on every particular molecular mechanism and signal transduction, which require further explorations into the potential links among the listed mechanisms. This provides novel insights into the understanding of SICD.

## Introduction

Sepsis is defined as a highly heterogeneous syndrome that is associated with a dysregulated systemic inflammatory host response to infection and causes organ dysfunction (Singer et al., 2016; Cecconi et al., 2018). Sepsis can be caused by bacterial, viral, and fungal infections and is a major cause of death in critical care patients. Damage-associated molecular patterns (DAMPs), which can be released by necrotic cardiomyocytes, can also induce potent inflammatory responses and cause sepsis-induced injuries. In-hospital mortality among patients with septic shock is reported to reach 40% (Singer et al., 2016). Septic shock—a series of circulatory, metabolic, and cellular abnormalities—is defined by a requirement for vasopressor support and persistent hyperlactatemia in the absence of hypovolemia (Gotts and Matthay, 2016; Hollenberg and Singer, 2021). Epidemiological studies showed that approximately 28.3–41% of all hospitalized patients with sepsis died due to multiple-organ failure (Levy et al., 2012). Furthermore, sepsis-induced cardiovascular dysfunction (SICD) was identified as being closely associated with higher mortality rates (Hochstadt et al., 2011; Romero-Bermejo et al., 2011). Cardiac dysfunction is one of the major complications of sepsis and is therefore predictive of a poor clinical outcome. The pathophysiological changes that occur during sepsis mean cardiac lesions might be induced by a series of factors including myocardial ischemia, myocardial depressant substance, inflammation, deregulation of adrenergic pathways, calcium overload, mitochondrial disorder, coronary microvascular dysfunction, and myocardial damage (Hollenberg and Singer, 2021). Animal and cell experiments with lipopolysaccharide (LPS)-induced sepsis models demonstrated a significantly higher rate of cardiomyocyte apoptosis, accumulation of intracellular reactive oxygen species (ROS), elevated cytoplasmic levels of cytochrome C, and activated inflammatory pathways (Han et al., 2020).

At present, there are more than 100 recognized modifications involved in regulating RNA stability and function. In eukaryotes, 5′ caps and 3′ poly-A modifications play an important role in transcriptional regulation, while mRNA internal modifications are used to maintain mRNA stability (Tong et al., 2018a). Methylation of the sixth nitrogen atom on the RNA molecule adenosine, termed N6-methyladenosine (m6A) modification, contributes to RNA splicing, translation, stability, and epigenetic effects among mRNAs and non-coding RNAs. Environmental exposures can alter the m6A modification levels in several signaling processes, and imbalances in m6A can mediate disease pathogenesis, providing novel insights into the potential application of m6A modifications as biomarkers for numerous diseases, immune responses, and autoimmunity pathogenesis (Li et al., 2017; Tong et al., 2018b). In addition, m6A participates in regulating bacterial infection according to an analysis of m6A-SNP and expression quantitative trait locus (eQTL) data. Sun et al. identified 1,321 genes as locations of m6A-cis-eQTLs (Sun et al., 2020). These genes were enriched in pathways of platelet degranulation and *Staphylococcus aureus* infection, which are vital for the pathophysiological process of sepsis. Collectively, this emerging evidence indicates a convincing association between m6A modification and sepsis attacks. Moreover, such regulation of RNA methylation has been shown to be involved in several types of cardiovascular diseases. This review summarizes the relative evidences available to support whether m6A modification facilitates the mechanisms between sepsis-induced immune responses and cardiomyopathy or myocardial injuries.

## N6-Methyladenosine Modification in Cardiovascular Diseases

**FIGURE 1:**
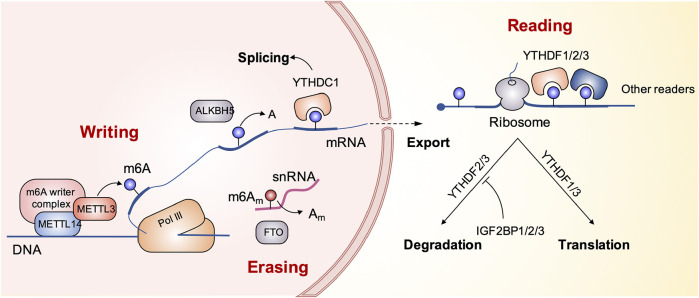
The schematic diagram presents the biological process of m6A modification.

The methylation of m6A is dynamically regulated by three regulatory factors, namely, methyltransferase (writers), demethylase (erasers), and methylated reading protein (readers) (Cao et al., 2016). Methylation of m6A is catalyzed by a multi-component methyltransferase complex consisting primarily of methyltransferase-like 3 (METTL3), methyltransferase-like 14 (METTL14), and WT1-associated protein (WTAP). METTL3 plays a central role and forms a dimer complex with METTL14 at a similar ratio, which is mainly located in the nuclear macular region. M6A-methylated mRNA requires a specific RNA-binding protein, methylated reader, to perform a specific biological function. The discovery of demethylase showed that the modification of m6A methylation was reversible. Currently known demethylation enzymes include FTO and ALKBH5, both of them belong to the ALKB family. The reading proteins found so far include YTH domain proteins (including YTHDF1, YTHDF2, YTHDF3, YTHDC1, and YTHDC2), the heterogeneous nuclear ribonucleoprotein family HNRNP (including HNRNPA2B1 and HNRNPC), and the IGF2BPS family (including IGF2BP1, IGF2BP2, and IGF2BP3), which are involved in mRNA translation, degradation, and processing (Li et al., 2017; Gao et al., 2020; Chen et al., 2021a; Chao et al., 2021; Zhou et al., 2021). The schematic diagram in Figure 1 presents the biological process of m6A modification.

To retrieve evidence on the relationship between m6A and SICD, it is important to identify the essentiality of m6A in cardiovascular diseases, which demonstrates that such RNA modification contributes significantly to the biological functions of heart contraction, coronary artery perfusion, and energy production. This section summarizes the evidence for the involvement of m6A modification in cardiovascular dysfunctions (CVDs) based on clinical research studies of patients with CVDs and basic biology explorations using animal or cell models of CVDs. The essential role of m6A in heart development and the generation of cardiomyopathy was revealed from such studies.

Regulation of heart development is extremely complex. The cardiovascular system consists of cardiomyocytes, fibroblasts, pericytes, endothelial cells, vascular smooth muscle cells, and cardiac-resident macrophages, which are all heterogeneous, and cells of the same type even show heterogeneity in different locations. m6A was found to play an important role in the regulation of heart development. Batista et al. analyzed the embryonic stem cell (ESC) m6A methylome in mouse and human cells and discovered extensive m6A modification of ESC genes, including most key regulators of ESC pluripotency and lineage control (Batista et al., 2014). Genetic inactivation or depletion of mouse and human METTL3 led to m6A erasure on selected target genes, prolonged NANOG expression upon differentiation, and disrupted exit of ESCs from self-renewal towards differentiation into several lineages *in vitro* and *in vivo*.

Postnatal cardiomyocyte morphology and functional maturation also involve m6A. Transcriptome-wide mapping of m6A in mRNA allowed m6A targets in human and murine adult hearts to be cataloged. Increased m6A methylation was found in human cardiomyopathy as knockdown and overexpression of the m6A writer enzyme METTL3 affected cell size and cellular remodeling both *in vitro* and *in vivo* (Kmietczyk et al., 2019). Furthermore, Dorn et al. found that METTL3-mediated methylation of mRNA on N6-adenosines was a dynamic modification that was enhanced in response to hypertrophic stimuli and was necessary for a normal hypertrophic response in cardiomyocytes (Dorn et al., 2019). Cardiac-specific METTL3 knockout mice also exhibited morphological and functional signs of heart failure with aging and stress. Berulava et al. observed that changes in m6A RNA methylation exceeded changes in gene expression both in mice and humans during progression to heart failure. The RNAs with altered m6A RNA methylation were mainly linked to metabolic and regulatory pathways, while changes in RNA expression level predominantly represented changes in structural plasticity (Berulava et al., 2020). These studies show the role of the regulation of m6A methylation in the occurrence and development of cardiomyopathy, highlighting the critical importance of this stress response mechanism for maintaining normal heart function.

The above information support the idea that m6A modification is involved in many kinds of cardiomyocyte injuries. However, as there is limited direct evidence available for SICD, a general understanding of the cellular and molecular mechanisms of SICD is required to determine whether m6A modification potentially affects SICD based on indirect evidences.

## Mechanisms of Immune Regulation and Metabolism in Sepsis-Induced Cardiovascular Dysfunction

Under normal conditions, a controlled cellular response to bacterial products protects the host from infection. In sepsis, hyperactivation of the immune response leads to excessive production of various proinflammatory cytokines and induces cellular injury. In mammals, the innate immune system is the first line of host defense involved in detecting diverse invading microbial pathogens. Receptors of the innate immune system are activated by microbial components such as LPS, which is a key molecule involved in the initiation of sepsis syndrome. Cardiac dysfunction and cardiovascular collapse during sepsis result from increased levels of several cytokines and from increased production of nitric oxide (NO), which is cytotoxic and can form a complex by combining with *cis*-aconitase in the respiratory chain, mitochondrial respiratory chain complexes I and II, and the active site of key enzymes in DNA synthesis, thus destroying their activity and causing myocardium apoptosis, and mitochondrial failure in cardiomyocytes (CMs), which leads to further DNA damage and ATP depletion, resulting in secondary energy failure. Cardiomyocyte hypocontractility also occurs, which is related to hibernation to maintain myocyte viability by limiting oxygen consumption, energy requirements, and ATP. Moreover, it is reported that cardiac microvascular dysfunction is associated with cardiac dysfunction in patients with sepsis. Cardiac microvascular endothelial cells (CMVECs), accounting for one-third of all heart cells, exert an important influence on the normal condition of coronary microvessels and adjacent cardiomyocytes. The increased function of microvessels could induce hypoxic stress due to localized microcirculation failure, which might result in ischemic injuries and diffused fibrosis. Thus, maintenance of normal microvessel-based circulation is also an important aspect to preventing SICD.

### Toll-Like Receptors

Mammalian Toll-like receptors (TLRs) are pattern recognition receptors (PRR) that act as CD-14-associated signal transducers to help cells recognize and differentiate pathogens and initiate appropriate signaling cascades (Lackner et al., 2020). TLRs also bridge innate and adaptive immunity by inducing various costimulatory and effector molecules. TLR-1, 2, 4, 5, and 6 are expressed on the cell surface and activate the expression of pro-inflammatory cytokines (such as IL-1, IL-2, IL-6, IL-12, TNF-α, etc.) and cell surface adhesion molecules (such as ICAM-1, VCAM-1, and P-selectin) through nuclear factor kappa B (NF-κB) and interferon (IFN) signaling (Li et al., 2019; Ye et al., 2020).

TLR4 is the most studied TLR family member in SCID research (Chang et al., 2021). Studies on TLR4-deficient mice confirmed the essential role of TLR4 in mediating neutrophil migratory phagocytic functions, attenuating inflammation, reducing ROS generation, and enhancing bacterial clearance. In sepsis, LPS binds to the LPS-binding proteins MD-2 and CD14 and thus binds to TLR4. This interaction can lead to both MyD88-dependent and MyD88-independent signaling events (Feng et al., 2014). MyD88-dependent signaling pathways include activation of TLR4, which leads to recruitment of the adaptor protein MyD88 through its own TIR domain. This association leads to autophosphorylation of IRAK through homophilic interactions in the death domain (Gao et al., 2015). TNF receptor-associated factor 6 (Traf6) is then obtained to signal the activation of the protein kinase NF-κB to induce the kinase, which subsequently activates the IKB kinase to phosphorylate I-kB (Ma et al., 2016; Ren et al., 2016). This phosphorylation step promotes translocation of transcription factor NF-κB to the nucleus. However, TLR2 also increased cardiodepressant cytokine levels in the myocardium and serum and weakened the neutrophil migratory function, which sharpened the SICD (Zou et al., 2011; Zuo et al., 2020). TLR3 was observed to play a deleterious role in mediating cardiac dysfunction in sepsis by increasing cecal ligation and puncture-induced cardiomyocyte apoptosis as well as Fas and Fas ligand expression in the myocardium (Gao et al., 2012; Kalbitz et al., 2016; Fattahi et al., 2017).

### Cytokines

Cytokines are known to induce the release of additional inflammatory factors such as prostanoids, NO, and many others, which eventually contribute to myocardial dysfunction. The main inflammatory mediators that might contribute to SICD are TNF-α, IL-1β, and IL-6. These cytokines significantly depressed cardiac contractility activity *in vitro* (Chen et al., 2021b). Moreover, increased circulating serum levels of IL-6 were associated with the severity of illness and the degree of vasopressor requirement in patients with septic shock (Gamkrelidze et al., 2014).

### Endothelial Dysfunction

Sepsis induces endothelial abnormalities, which also lead to SICD. In response to inflammatory cytokines during sepsis, endothelial cells secrete more adhesion molecules, which increase leukocyte–endothelium interactions and facilitate neutrophilic infiltration that is damaging to cardiomyocytes (Lin et al., 2020a; Tu et al., 2020; Cao et al., 2021). The activated monocytes and macrophages are recruited by pro-inflammatory cytokines and are binding to ICAM-a and VCAM-1 molecules (Kang et al., 2012; Franceschelli et al., 2017). Subsequently, these monocytes can migrate into the inter-space among endothelial cells where they differentiate into type I macrophages causing fibrosis and endothelial damage (Li et al., 2012).

### Mitochondrial Dysfunction and Oxidative Stress

The heart is rich in mitochondria, which are involved in both energy provision and intracellular calcium regulation; consequently, the degree of mitochondrial dysfunction is tightly linked to SICD and the prognosis of this condition (Lin et al., 2020a; Shi et al., 2021a; Wang et al., 2021a). The activities of complexes I and II of the mitochondrial respiratory chain were diminished in the hearts from animals with sepsis (Piel et al., 2007; Neviere et al., 2016), and this might be due to the detrimental effects of sepsis mediators such as NO, TNF-α, IL-1β, etc. In addition, ROS accumulation can suppress mitochondrial function by destroying mitochondrial proteins, lipids, and DNA through structural modifications during sepsis in a positive feedback fashion as failed mitochondria led to significantly slower ROS scavenging (Potz et al., 2016). Oxidative stress can cause dynamic m6A modification in the 5′ UTR and 5′ vicinity of coding sequences (CDSs), which might play a vital role in overcoming the stress condition.

## N6-Methyladenosine Modification in Mediating Inflammation Responses

In recent years, m6A modification was shown to be involved in several inflammation-induced diseases, such as rheumatoid arthritis and diabetic retinopathy, and was confirmed to be associated with clinical prognosis of such diseases. Zhang et al. analyzed the gene expression datasets of 479 consecutive patients with severe sepsis and found ALKBH5, HNRNPC, KIAA1429, WTAP, and YTHDF2 were significantly correlated with 28-days cumulative mortality (*p* < 0.05). ALKBH5 and WTAP are risky genes with a hazard ratio (HR) > 1, while HNRNPC, KIAA1429, and YTHDF2 are protective genes with a HR < 1 (Zhang et al., 2020a). Lu et al. demonstrated that METTL3, METTL14, ALKBH5, FTO, and YTHDF2 participated in the inflammation activity in LPS-induced liver injuries (Lu et al., 2018).

m6A is also involved in the immune response in tumor growth and virus infection beyond sepsis attacks. Zhu et al. detected dozens of m6A-SNPs as critical functional polymorphisms and novel genetic biomarkers for ischemic stroke susceptibility and provided a new means of elucidating the biological mechanism underlying vascular inflammation development (Zhu et al., 2021). Chokkalla et al. presented the m6A epitranscriptome of the brain post-stroke, further verifying that m6A modification participated in inflammatory responses (Chokkalla et al., 2019). Recently, Li et al. identified two independent m6A modification patterns with distinct biological functions, immunological characteristics, and prognoses in renal clear cell carcinoma and showed that low-m6A-score groups reflected an inflammatory phenotype, which may be more sensitive to anticancer immunotherapy (Li et al., 2021a). Emerging research indicates that depletion of the host cell m6A methyltransferase METTL3 decreases m6A levels in SARS-CoV-2 and host genes, and the reduction of m6A in viral RNA increases RIG-I binding and subsequently activates downstream innate immune signaling pathways and inflammatory gene expression (Li et al., 2021b; Qu et al., 2021).

Current evidence clearly supports a relationship between m6A modification and inflammation syndrome(s). As sepsis is considered as acute and severe inflammation attacks, it is believed that m6A regulation participates in SICD. Therefore, the following subsections summarize the “writer,” “eraser,” and “reader” in the regulation of cytokines, monocytes/macrophages, and related non-coding RNAs in inflammation.

### “Writer” and Inflammation

#### Cytokines

METTL3 mediated the increased expression of inflammatory cytokines, such as IL-6 and IL-8, and the activation of NF-κB signaling under immune stimulations (Sang et al., 2021). Mechanistically, the promoted expression of cytokines is induced by activation of Traf6 (Wen et al., 2020), and the inflammatory response is suppressed by METTL3 ablation. Knockdown of METTL3 decreased the expression of inflammatory cytokines and phosphorylation of IKKα/β, p65, and IκBα in NF-κB signaling. The depletion of METTL3 attenuated the MAPK pathways involving p38, ERK, and JNK. METTL3 knockdown facilitated the expression of MyD88S, a splice variant of MyD88 that inhibits inflammatory cytokine production, suggesting that METTL3 might inhibit the LPS-induced inflammatory response by regulating alternative splicing of MyD88 (Feng et al., 2018). MyD88 acts as a shared adaptor molecule for most TLRs and can elicit the production of multiple inflammatory cytokine genes and the activation of NF-κB and MAPK involved in neurotoxicity. The depletion of METTL3 decreased the m6A level of Traf6 mRNA, thereby its transcripts were trapped in the nucleus, and the decreased expression of Traf6 led to the suppression of NF-κB and MAPK signaling pathways (Zong et al., 2019). Besides, METTL3 knockdown promoted mRNA expression and stability of negative regulators of Smad signaling, Smad7 and Smurf1 (Zhang et al., 2019). Thus, METTL3 depletion inhibited proinflammatory cytokine expression by alleviating Smad signaling pathways.

METTL14 promoted FOXO1 expression *via* increased levels of m6A modification and induced endothelial cell inflammatory responses by facilitating TNF-a, IL-1, IL-3, and IL-6 secretion (Jian et al., 2020). Zong et al. revealed that the interplay between FOXO6 and METTL3 regulated the m6A level of GPR161 signaling and the expression of β-defensin during *Escherichia coli* infection (Zong et al., 2021a). The re-expression of METTL3 facilitated defensin accumulation (Zong et al., 2021b). The dissociation of METTL3 from the METTL3–METTL14–WTAP complex contributed to decreased miR-92b-3p expression in an m6A-dependent post-transcriptional modification manner and increased the protein level of the phosphatase and tensin homolog (PTEN), which subsequently inactivated the PI3K/AKT signaling pathway (Lin et al., 2020b). The PI3K/AKT pathway is known to inhibit FOXO1, which regulates endothelial immune responses.

#### Monocytes/Macrophages

Monocytes can be activated and recruited by the release of inflammatory cytokines. Activated monocytes adhere to endothelial cells to cause cellular injuries, then the monocytes differentiate into macrophages and migrate into the vascular smooth muscle layer, which leads to fibrotic changes to coronary arteries, as well as damage to myocardial tissues. Therefore, the biological functional status of monocytes or macrophages also greatly contributes to the progress of SICD. Through the use of a pooled CRISPR screening strategy, Tong et al. determined that transcripts of the *Irakm* gene, encoding a negative regulator of TLR4 signaling, were highly decorated by m6A modification, and congruent with this, Mettl3^flox/flox^;Lyzm-Cre mice displayed increased susceptibility to bacterial infection (Tong et al., 2021). METTL3 deficiency led to the loss of m6A modification on *Irakm* mRNA and slowed its degradation, resulting in an increased amount of IRAKM, which ultimately suppressed TLR signaling-mediated macrophage activation (Tong et al., 2021). Moreover, IFN regulatory factor-1 (IRF-1) induced METTL3 expression in macrophages and inhibited the expression of circ_0029589, triggering macrophage pyroptosis (Guo et al., 2020). A controversial opinion was proposed that METTL3 mediated the activities of macrophages in response to LPS stimulation *via* NF-κB signaling and that the overexpression of METTL3 could significantly attenuate the inflammatory response induced by LPS in macrophages (Wang et al., 2019a).

METTL14 in myeloid cells exacerbated macrophage responses to acute bacterial infection in mice, which led to higher mortality. METTL14 reduced the rate of Socs1 m6A methylation and decreased YTHDF1 binding. The reduced YTHDF1 m6A modification led to diminished SOCS1 and over-activated TLR4/NF-κB signaling. Overexpressing SOCS1 under METTL14 or YTHDF1 rescued the phenotype of macrophage activation *in vitro* and *in vivo*. Accordingly, FTO showed an antagonistic role of METTL14 in regulating macrophage activity (Du et al., 2020). Besides, METTL14 interacted with FOXO1 and acted directly on the promoter regions of VCAM-1 and ICAM-1, which enhanced monocyte binding to endothelial cells during atherosclerosis (Jian et al., 2020). METTL14 also regulated the expression of AKT signaling, and a METTL14 knockout exhibited reduced inflammation and cell survival behavior (Liu et al., 2019a).

#### miRNAs

Non-coding RNAs, which are involved in essential biological processes, are also subject to methylation by m6A modification. METTL3 has a major catalytic role in promoting miR-21-5p maturation, and miR-21-5p could activate the SPRY1/ERK/NF-kB pathway, which is involved in inflammation responses leading to renal fibrosis (Liu et al., 2021a). METTL3 positively modulated miR-365-3p processing in a microprocessor protein DiGeorge critical region 8-dependent manner, inducing inflammation and pain behavior. miR-365-3p was verified to be involved in cardiac hypertrophy (Zhang et al., 2020b). The activity of m6A modification was also regulated by other mechanisms because SUMOylation of METTL3 regulated its m6A RNA methyltransferase activity without altering its stability, localization, or interaction with METTL14 and WTAP (Du et al., 2018).

#### “Erasers” and Inflammation

Opposite to the writer function, eraser leads to demethylation of RNA in an m6A modification manner. Therefore, understanding the eraser function in controlling inflammation is also necessary. ALKBH5 upregulated the expression of MALAT1 by demethylation, which participates in the production of inflammatory cytokines in HK-2 cells under LPS stimulation (Zhu and Lu, 2020). In addition, dexamethasone inhibited ALKBN5, which decreased related inflammatory cytokines and induced cell apoptosis (Zhu and Lu, 2020). ALKBH5 also participated in demethylating m6A residues in the 3′ untranslated region (UTR) of high-mobility group box 1 (HMGB1) (Chen et al., 2021c). This demethylation resulted in the activation of STING-IRF3 signaling, which mediated the innate immune response in radiation-induced liver disease (Chen et al., 2021c). CircZbtb20 enhanced the interaction of ALKBH5 with Nr4a1 mRNA, leading to ablation of the m6A modification of Nr4a1 mRNA to promote its stability (Liu et al., 2021b). Nr4a1 initiated Notch2 signaling activation, which contributed to the maintenance of type 3 innate lymphoid cell homeostasis. The deletion of ALKBH5 or Nr4a1 also impaired ILC3 homeostasis and increased susceptibility to bacterial infection (Liu et al., 2021b). Zhao et al. found that silencing zinc finger and BTB domain-containing protein 7B (Zbtb7b) resulted in higher radiation-induced IL-6 production in THP1 cells and BEAS-2B lung bronchial epithelial cells. Zbtb7b recruited RNA demethylase ALKBH5 to IL6 mRNA (Zhao et al., 2020). Subsequently, ALKBH5 demethylated the m6A modification of IL6 mRNA and inhibited its nuclear export in endothelial cells.

FTO has increasingly been found in response to inflammation. FTO regulates the integrin signaling pathway and inflammation signaling pathways. An RNA decay assay revealed that TEAD2 mRNA stability was impaired by FTO because FTO stimulated the degradation of TEAD2 and inhibited the formation of the YAP–TEAD complex, a main factor downstream of integrin signaling (Rong et al., 2019). YAP participates in TLR2 and TLR4 regulation, showing an anti-inflammatory function (Gao et al., 2021). In addition, Yu et al. found that an FTO-mediated YTHDF2 epigenetic modification increased the rate of methylation of PPARα, leading to activation of NLRP3 and NF-κB-driven inflammation (Yu et al., 2021). FTO also helped disable the mRNA of pro-inflammatory genes to suppress the activity of inflammatory responses, including IL-1β and TNF-α. In addition, SIRT1 activated RANBP2, which is indispensable for FTO SUMOylation at K-216 sites, leading to destabilization of FTO (Liu et al., 2020a).

### “Readers” and Inflammation

#### YTHDF1

YTHDF1 mediated the translation of XPO1 in an m6A-dependent manner by methylating the 5′ UTR of XPO1 RNA. The methylated XPO1 RNA provided greater amounts of XPO1 protein, which resulted in NK-κB activation and subsequent inflammation (Olazagoitia-Garmendia et al., 2021). A report from Zong et al. provided evidence on the function of YTHDF1 during infection of intestinal epithelial cells. The P/Q/N-rich domain in YTHDF1 was found to interact with the DEAD domain in the host factor DDX60, which helped target the Traf6 mRNA and regulated m6A modification near the stop codon of the transcript to initiate the expression of Traf6 (Zong et al., 2021a). The depletion of YTHDF1 alleviated the immune response to bacterial infection as evidenced through an observation that YTHDF1 knockdown macrophages improved the immune paralysis of macrophages, Th1/Th17 cells, and cytotoxic T lymphocytes and reduced the entry of macrophages into the brain to cause endothelial damage in severe sepsis rats with ECMO (Xing et al., 2021). This was through the inhibition of HMGB1/RAGE and YTHDF1 and m6A RNA methylation of JAK2/STAT3 in macrophages (Xing et al., 2021). The overexpression of miR-421-3p inhibited the expression of YTHDF1, which mediated the m6A modification of p65 mRNA. YTHDF1 is known to enhance the expression of p65; thus, miR-421-30/YTHDF1 presented a suppressing role on pro-inflammation factors (Zheng et al., 2020).

#### YTHDF2

Experimentally induced inflammation increased YTHDF2 expression. YTHDF2 ablation increased the absence of multiple m6A-targeted inflammation transcripts, including IFN-γ, TNF-α, IFN-α, IFN regulatory factor 7, STAT1, and TLR4 signaling pathways, which led to the activation of pro-inflammation pathways (Mapperley et al., 2021). Moreover, YTHDF2 processed the decay of m6A containing the LPS-induced IL-1β, IL-6, IL-11, and IL-12 expression and the phosphorylation of p65, p38, and ERK1/2 in NF-κB and MAPK signaling. YTHDF2 depletion increased the expression and stability of MAP2K4 and MAP4K4 mRNAs (Yu et al., 2019). Together, these results suggest that YTHDF2 knockdown increases mRNA expression levels of MAP2K4 and MAP4K4 *via* stabilization of the mRNA transcripts, which activates MAPK and NF-κB signaling pathways, promoting the expression of proinflammatory cytokines and aggravating the inflammatory response in LPS-stimulated cells.

The loss of YTHDF2 promoted demethylation of H3K27me3, which led to an enhanced production of proinflammatory cytokines. The mRNA of lysine demethylase 6B (KDM6B) was m6A-modified, and its decay was mediated by YTHDF2. YTHDF2 deficiency stabilized KDM6B to promote H3K27me3 demethylation of multiple proinflammatory cytokines and subsequently enhanced their transcription (Wu et al., 2020). Besides, CCR7 stimulation upregulated lnc-Dpf3 *via* removal of the m6A modification to prevent RNA degradation by inhibiting YTHDF2. Lnc-Dpf3 directly bound to HIF-1α and suppressed HIF-1α-dependent transcription of the glycolytic gene *LDHA*, thus inhibiting dendritic cell (DC) glycolytic metabolism and migratory capacity. DC-specific lnc-Dpf3 deficiency also increased CCR7-mediated DC migration, leading to exaggerated adaptive immune responses and inflammatory injuries, which indicated that YTHDF2 had the capability to inhibit lnc-Dpf3 expression to enhance inflammatory activity (Liu et al., 2019b). Lysine acetyltransferase 1 (KAT1) activated the transcription activity of YTHDF2 through histone acetylation of the promoter. YTHDF2 then triggered the instability of ITGB1 mRNA to induce mRNA degradation in an m6A-dependent manner and silenced the FAK–PI3K–AKT pathway in endothelial cells, which alleviated the interaction between monocytes and endothelial cells (Qi et al., 2021). Also, SUMOylation of YTHDF2 had little impact on its ubiquitination and localization, but significantly increased its binding affinity to m6A-modified mRNAs and subsequently resulted in deregulated gene expression (Hou et al., 2021).

#### Others

Besides the YTH domain family, several other readers are involved in m6A modification under inflammatory responses. Molecular studies indicated that IGF2BP2 switched M1 macrophages to M2 activation by targeting tuberous sclerosis 1 *via* an m6A-dependent process. The depletion of IGF2BP2 also enhanced the M1 phenotype. Additionally, it also has a mediator function on PPARγ involved in the differentiation of M2 macrophages (Wang et al., 2021b). Bechara et al. demonstrated the unexpected involvement of m6A modification in regulating C/EBPβ and C/EBPδ under autoimmune inflammation. The reader “IMP2” facilitated IL-17-mediated Cebpd mRNA stabilization and promoted translation of C/EBPβ/δ. In addition, Imp2 (−/−) mice were resistant to autoantibody-induced glomerulonephritis (AGN), showing impaired renal expression of C/EBPs and LCN2 (Bechara et al., 2021).

## N6-Methyladenosine Facilitates Mitochondrial Dysfunction

### Mitochondrial Biogenesis

The biogenesis of mitochondria is critical for maturation of mitochondrial function. Sufficient amounts of mitochondria help maintain cardiomyocyte morphology and optimal energy production (Liao et al., 2020). METTL3-mediated RNA m6A modification and activation of mitochondrial fusion proteins MFN2 and OPA1, as well as inhibition of the mitophagy BNIP3/NIX receptor pathway, restored mitochondrial function. ALKBH1-deficient HEK293 cells showed increased mtDNA copy numbers and mitochondrial dysfunction (Lv et al., 2021). Meanwhile, Du et al. found that the expression of m6A demethylase FTO was decreased during ischemia–reperfusion injury. FTO contributed to the protective effect *via* demethylation of the Drp1 mRNA and impairment of the Drp1-mediated mitochondrial fragmentation. LPS-induced sepsis caused a continuous high expression of Drp1 (Du et al., 2021), and Drp1 induced mitochondrial fragmentation leading to mitochondrial dysfunction. In addition, AAV-delivered FTO could ameliorate the ischemia–reperfusion injury, repress the elevated level of m6A-methylated RNA, and alleviate oxidative stress and mitochondrial fragmentation *in vivo* and *in vitro* (Liu et al., 2020b). Moreover, a research from Wang et al. demonstrated that downregulation of FTO suppressed mitochondrial biogenesis and energy production, shown as decreased mitochondrial mass and mtDNA content through inhibition of PGC-1α expression. Importantly, this research revealed that this regulation was mTOR signaling-dependent. The suppression of mTOR blocked the enhanced transcription of FTO-induced PGC-1α (Wang et al., 2017). Readers also participate in maintaining mitochondrial biogenesis. The overexpression of either YTHDF1 or YTHDF2 led to the upregulation of the expression of MGMT, NDRG1, PGC1, LONP1, and TFAM, resulting in a more stable mitochondrial homeostasis (Ozkurede et al., 2019).

### Mitochondrial Metabolism

Besides the morphology of mitochondria, the metabolic function or status is related to the prognosis of cardiovascular diseases. Thus, deciphering the relationship between m6A modification and mitochondrial function is critical to reveal the potential mechanisms of m6A in SICD. METTL3 regulated NRIP1 (encoding RIP140) expression in an m6A-dependent manner, and METTL3 knockdown reduced the m6A modification of NRIP1 mRNA and increased its expression. The decay rate of NRIP1 mRNA was significantly reduced in METTL3 knockdown cells but increased in METTL3-overexpressing cells. RIP140 has a role in inflammation since it acts as a coactivator for NF-κB/RelA-dependent cytokine gene expression. Lack of RIP140 leads to inhibition of proinflammatory pathways in macrophages. RIP140 is also instrumental in regulating lipid and glucose metabolism and regulates gene expression in metabolic tissues including the heart and skeletal muscle (Shi et al., 2021b). NRIP1 affects oxidative metabolism and mitochondrial biogenesis by negatively controlling mitochondrial pathways regulated by PGC-1α. The downregulation of NRIP1 would thus attenuate the dysfunction of mitochondria. However, a study by Zhuang et al. reported that FTO enhanced the expression of PGC-1α, which helped restore mitochondrial function (Zhuang et al., 2019).

Adenylate kinase 4 (AK4) participates in the regulation of the ADP/ATP production cycle of mitochondria. However, AK4 was identified as being downregulated in sepsis, which lowered the cellular ATP level (Liu et al., 2020c). Liu et al. demonstrated that METTL3 controlled the m6A modification of AK4 mRNA and maintained the expression of the protein. A higher level of expression of AK4 promoted inflammatory gene expression *via* Hif-1α and AMPK signaling in macrophages and induced ROS accumulation (Liu et al., 2020c). Moreover, the transcription factor Hif-2α was mediated by METTL3 methylation, and this enhanced its expression. Hif-2α then targeted MTHFD2 and induced MTHFD2 gene transcription. MTHDF2 is involved in the serine metabolism circle and supported IL-1β production in macrophages (Li et al., 2020).

### Mitochondrial Apoptosis

m6A modification marks mitochondrial stress response genes and promotes their transcription to alleviate mitochondrial stress in progeny. MiR-600 inhibited the expression of METTL3 and increased the Bax/Bcl-2 ratio, indicating activation of the mitochondrial apoptotic pathway (Wei et al., 2019). Jia et al. revealed that YTHDF1 promoted the translation of methylated HINT2 mRNA, which worked as a mitochondrial apoptotic sensitizer to induce cell death (Jia et al., 2019). Furthermore, Shen et al. demonstrated that FTO suppressed the mitochondrial unfolded protein response (UPRmt) and inhibited the activation of caspase-3 *via* the JAK2/STAT3 signaling pathway. UPRmt is associated with mitochondrial function. In septic rats, UPRmt is known to be downregulated under sepsis attacks (Shen et al., 2021). Enhancement of endogenous UPRmt through oligomycin administration reduced sepsis-mediated mitochondrial injury and myocardial dysfunction through mitophagy (Wang et al., 2021a).

## N6-Methyladenosine Maintains Endothelial Homeostasis

### Participation in Coronary Artery Diseases

m6A methylation is the most prevalent post-transcriptional modification on mRNAs. Post-transcriptional regulation has several advantages, such as prompt stimuli responses, fine-tuning of protein amounts, and localized regulation control. In coronary heart disease, endothelial dysfunction caused by various factors and the repair after hypoxia stimulation play an important role in pathological changes.

Firstly, METTL3 was shown to be involved in plaque formation by stimulating recruitment of monocytes and macrophages. Altered m6A levels were confirmed in leukocytes and the endothelium in western diet-induced atherosclerosis mice and in oxidized-LDL (ox-LDL)-treated human endothelium and monocyte cells (Wu et al., 2019). The upregulation of METTL3 expression increased NF-κB and p65 phosphorylation and enhanced monocyte adhesion to endothelial cells (Chien et al., 2021). In addition, METTL3-mediated RNA hypermethylation upregulated NLRP1 transcripts and downregulated KLF4 transcripts through YTHDF1 and YTHDF2 m6A reader proteins, which trigger the atherogenesis (Chien et al., 2021). Another m6A writer, METTL14, has been shown to interact with FOXO1 and act directly on the promoter regions of VCAM-1 and ICAM-1, which enhanced the binding of monocytes to endothelial cells during atherosclerosis (Jian et al., 2020). Moreover, METTL14 increased the m6A modification of pri-miR-19a and promoted the processing of mature miR-19a, thus enhancing the proliferation and invasion of CMVECs (Zhang et al., 2020c). Moreover, miR-19a was shown to improve prognosis and clinical cardiac function after myocardial infarction (Gao et al., 2019).

Secondly, the demethylase (“eraser”) contributed to coronary artery diseases in a deteriorative role. ALKBH1 decreased the m6A modification of the myocardial infarction associated transcript (MIAT) promoter region, which allowed HIF-1α binding to this site to upregulate the transcripts of lnc-MIAT. In turn, lnc-MIAT promoted the inflammation response to sepsis in endothelial cells, driving endothelial dysfunction (Wu et al., 2019). Furthermore, Zhao et al. demonstrated that ALKBH5 was elevated during hypoxic stimulation of CMVECs and caused a general reduction in m6A modification. ALKBH5 attenuated the stability of the mRNA of WNT5A and incited the process of angiogenesis, which exacerbated the hypoxia of CMs during sepsis attacks. An AAV strategy has been used to knockdown ALKBH5 to promote angiogenic phenotypes (Zhao et al., 2021). Therefore, METTL3 and METTL14 worked in a protective role to maintain microvascular circulation and myocardial function during sepsis. Controversially, ALKBH1 presented a negative role in maintaining the normal biological function of endothelial cells.

### Involvement in Angiogenesis

METTL3 predominantly participates in the regulation of vascular endothelial growth factor (VEGF)-A, which is regulated by mTOR-AKT, NOTCH, and LRP6 signaling, as well as ubiquitylation modification. Studies from Tian et al. (Tian et al., 2019) and Chen et al. (Chen et al., 2019) demonstrated that knockdown of METTL3 also restrained the expression of VEGF-A. Mechanistically, deletion of METTL3 leads to the suppression of endothelial tyrosine kinase and VEGF-A through reduced abundance of m6A peaks on a specific region (Wang et al., 2021c). Parial et al. observed that knockdown of METTL3 in human endothelial cells inhibited cell proliferation, migration, and capillary tube formation. METTL3 was found to activate the phosphorylation of PHLPP2-mTOR-AKT signaling to enhance angiogenesis (Parial et al., 2021). Yao et al. studied the function of METTL3 in endothelial cells under hypoxia stress and found that METTL3 promoted neovascularization by methylating the mRNA of LRP6 and DVL1 in a YTHDF1-dependent manner. LRP6 and DVL1 were initiated in LPS-induced endothelial cells (Yao et al., 2020). METTL3 also increased splicing of precursor miR-143-3p to facilitate its biogenesis, and miR-143-3p bound to sites in the 3′ UTR of vasohibin-1 (VASH1) to inhibit its expression. A lower expression of VASH1 was presumed to reduce ubiquitylation of VEGF-A and increase VEGF-A expression (Wang et al., 2019b).

Two pioneering studies in 2017 and 2018 provided the initial understanding of how m6A regulates endothelial fate and function in angiogenesis (Zhang et al., 2017; Lv et al., 2018). Endothelial expression of METTL3 could methylate Notch1 mRNA, and this led to repression of Notch activity. Moreover, YTHDF2-mediated mRNA decay of the arterial endothelial genes *Notch1a* and *rhoca* also contributed to this maintenance of normal angiogenesis under METTL3 methylation (Zhang et al., 2017). In addition, METTL3 could reduce the level of heterodimeric Notch E3 ubiquitin ligase formed by DTX1 and DTX3L and continuously activate the Notch signaling pathway, which affected the angiogenesis of endothelial cells (Wang et al., 2020a).

Similarly, high levels of METTL14 expression on vascular endothelial cells (CD31^+^) suggested that angiogenesis was accompanied by differential modification of m6A RNA. However, miR-4729 inhibited the expression of METTL14 by targeting the 3′ UTR of METTL14 mRNA and reducing the m6A modification of TIE1 mRNA and downstream VEGF-A signaling (Liu et al., 2021c). A subsequent study confirmed that IGF2BP3 could bind to VEGF mRNA and read the m6A modification, thus regulating both expression and stability of VEGF mRNA. Knockdown of IGF2BP3 repressed angiogenesis (Yang et al., 2020). VEGF has also been reported to be increased in response to septic inflammation, which provides a reasonable explanation for the regulation of METTL3, METTL14, and IGF2BP3 in sepsis. In addition, WTAP maintains desmoplakin (DSP) expression through m6A modification in an IGF2BPS-dependent manner and enhances angiogenesis of endothelial cells *in vitro* by regulating WNT signaling activity (Wang et al., 2020b).

## Conclusion and Future Aspects

**FIGURE 2:**
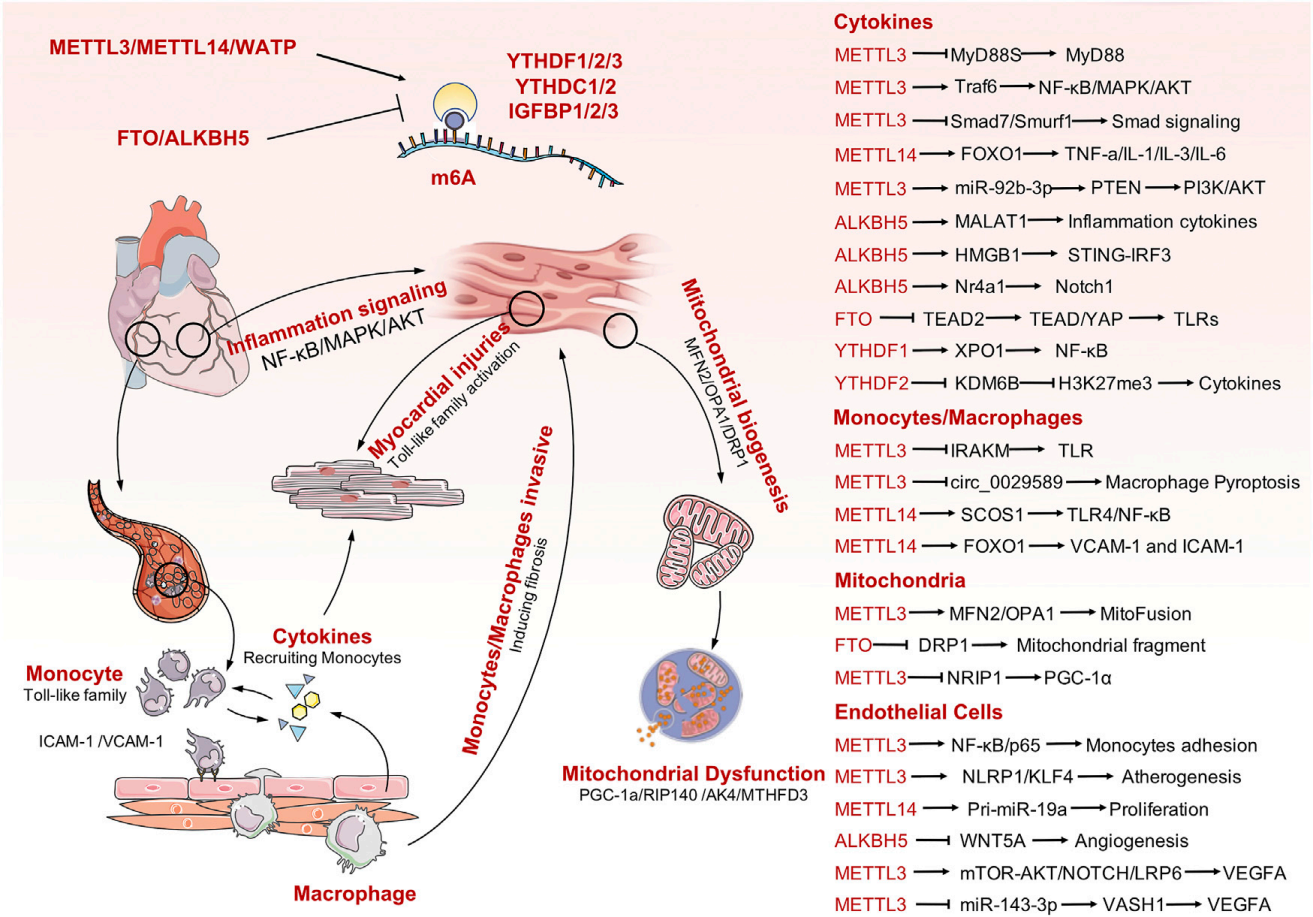
The mechanisms involved in SICD and the potential m6A modification process that would contribute to SICD. Some important m6A-mediating targets or signaling pathways are also listed in the right panel.

Sepsis is a life-threatening organ dysfunction caused by a host’s dysfunctional response to infection. Pathogenic bacteria invade the human heart, lungs, liver, spleen, and other organs and produce inflammatory substances in these tissues. This can result in various cardiovascular diseases. Septic heart disease occurs because pathogens invading the blood stimulate the activation of endothelial cells, cause accumulation of vast quantities of white blood cells, and trigger an immune response. During the immune response, numerous inflammatory factors are released into cells to participate in the inflammatory process, which ultimately damages cardiac myocytes and leads to impaired cardiac function. Mitochondrial dysfunction is one of the main manifestations of myocardial cell injury. Increasing evidence has demonstrated the role of m6A modification in multiple biological processes, particularly for sepsis and immune disorders. In addition, a series of studies have confirmed the role of m6A modification in the regulation of cardiovascular diseases. Unfortunately, there is limited evidence available to analyze the association between m6A modification and SICD. In this review, we firstly summarized current evidence on m6A mediation of the pathophysiological processes involved in cardiac development and cardiomyopathy to emphasize the importance of RNA methylation in maintaining heart biogenesis and homeostasis. Next, we clarified the participants of m6A modification in extended inflammation responses and immune system activation, which are the dominant and initial changes secondary to sepsis attack. Finally, the predominant causes of SICD were analyzed in detail, identifying activation of TLRs and inflammatory cytokines, endothelial cell dysfunction, and mitochondrial failure. Thus, this review systematically collates all related potential mechanisms between m6A modification and causes of SICD and provides novel insights into the understanding of SICD. Although there is lack of direct evidence available for SICD, the indirect evidences have been presented, case by case, for each particular molecular mechanism and signaling transduction process (Figure 2). Further explorations are required into the potential links among the listed mechanisms to obtain direct evidence for the roles of m6A modification in SICD.

## Abbreviations

AK4, adenylate kinase 4; CMs, cardiomyocytes; CMVES, cardiac microvascular endothelial cells; CVDs, cardiovascular dysfunctions; DC, dendritic cell; DSP, desmoplakin; eQTLs, expression quantitative trait loci; ESC, embryonic stem cell; HMGB1, high-mobility group box 1; IFN, interferon; IRF-1, IFN regulatory factor-1; KAT1, lysine acetyltransferase 1; LPS, lipopolysaccharide; m6A, N6-methyladenosine; NF-κB, nuclear factor kappa B; PRR, pattern recognition receptors; PTEN, phosphatase and tensin homolog; ROS, reactive oxygen species; SICD, sepsis-induced cardiovascular dysfunction; Traf6, TNF receptor-associated factor 6; UPRmt, mitochondrial unfolded protein response; UTR, untranslated region; VASH1, vasohibin-1; VEGF, vascular endothelial growth factor; Zbtb7b, zinc finger and BTB domain-containing protein 7B.
